# Hell Is Other People? Gender and Interactions with Strangers in the Workplace Influence a Person’s Risk of Depression

**DOI:** 10.1371/journal.pone.0103501

**Published:** 2014-07-30

**Authors:** Sebastian Fischer, Anita Wiemer, Laura Diedrich, Jörn Moock, Wulf Rössler

**Affiliations:** 1 Innovation Incubator, Leuphana University Lueneburg, Lüneburg, Germany; 2 Department of Psychiatry, Psychotherapy and Psychosomatics, Psychiatric Hospital, University of Zurich, Zurich, Switzerland; 3 Institute of Psychiatry, Laboratory of Neuroscience, University of Sao Paulo, Sao Paulo, Brazil; Harvard School of Public Health, United States of America

## Abstract

We suggest that interactions with strangers at work influence the likelihood of depressive disorders, as they serve as an environmental stressor, which are a necessary condition for the onset of depression according to diathesis-stress models of depression. We examined a large dataset (N = 76,563 in K = 196 occupations) from the German pension insurance program and the Occupational Information Network dataset on occupational characteristics. We used a multilevel framework with individuals and occupations as levels of analysis. We found that occupational environments influence employees’ risks of depression. In line with the quotation that ‘hell is other people’ frequent conflictual contacts were related to greater likelihoods of depression in both males and females (*OR* = 1.14, *p*<.05). However, interactions with the public were related to greater likelihoods of depression for males but lower likelihoods of depression for females (*OR_intercation_* = 1.21, *p*<.01). We theorize that some occupations may involve interpersonal experiences with negative emotional tones that make functional coping difficult and increase the risk of depression. In other occupations, these experiences have neutral tones and allow for functional coping strategies. Functional strategies are more often found in women than in men.

## Introduction

The classic quote ‘hell is other people’ from Sartre’s play ‘No Exit’ [1] alludes to the discomfort that most people occasionally feel when they are around strangers and cannot leave the situation. In the original play, the impossibility of escaping from human contact is symbolized by a peculiar vision of hell; i.e. a room in which strangers are locked together for eternity. Similarly, the scientific literature on service occupations has identified interpersonal contacts with non-familiar others, coupled with an inescapable situation, as a major stressor [2]–[4].

Frequent service interactions with strangers may even increase depressive symptoms and lead to more absence from work [5]. In this paper, we investigated gender differences in the relationship between interactions with strangers in the workplace and the likelihood of severe depression necessitating rehabilitation. We emphasized that the emotional tones that accompany every act of typical interactions at work are relevant to the onset of depression.

We focus on clinically diagnosed depression because this is the most frequent mental disorder among employees (6.6% 12-month prevalence rate in the US population [6]). Depression pervades a person’s entire life, and high rates of chronic depression [7] are related to increased unemployment and early retirement [6]. Social systems invest heavily in measures that seek to reintegrate depressed individuals into the labor market, which is a costly and time-consuming endeavor for the depressed patient, and full reintegration is difficult to achieve [8]. Nonetheless, reintegration should be the goal for those who suffer from depression, as it benefits society, employers and the depressed patient.

This problem of reintegration necessitates research on the risk factors of depression, with an emphasis on gender [9]. Epidemiological studies indicate that the prevalence and incidence of unipolar depressive disorders among women are approximately twice the rates among men [10].

### Understanding Workplace Characteristics in Relation to Rates of Depression

Aside from personal factors such as gender, diathesis-stress models of depression suggest that environmental stressors are a necessary condition for the onset of depression [7], [11]–[13]. The literature has reported that elevated rates of depression in some occupations are due to occupation-specific emotional stressors (e.g. [14]–[16]). Accordingly, occupations that involve frequent interactions with other people may entail higher rates of depression because these occupations involve strong emotional stressors.

Other authors have theorized that gender may influence rates of depression, as men and women handle emotional stressors differently, i.e. have different resources [17]–[19]. Women have been found to handle emotions with more functional strategies than men [20], [21]. For example, they display emotions more openly but also avoid negative emotions through withdrawal, compromise or indirect actions instead of direct, assertive actions, which are suggested to be more prominent in males [19]. In contrast, dysfunctional handling of emotions (e.g. suppression of emotions [18]) has been found to be related to increased subjective ill-health [22], [23]. This makes females better suited for people-oriented occupations. However, recent studies have shown that females seem to use *both* positive and negative strategies more often than men [24], and this finding may be interpreted to indicate that females engage in emotional work more often than men [24], may derive more benefit from emotional work [25], or may be more sensitive to environmental factors, which would increase the necessity of emotion regulation [26]. Similarly, females have been suggested to be more sensitive to others’ emotions (i.e., more emotionally intelligent; [27]).

Conflating these results, the literature has generally concluded that work environments influence the onset of depression and that gender differences in the handling of interpersonal emotional stressors may be related to gender differences in the onset of depression. It seems incongruent that, although females have a higher average prevalence of depressive disorders and also work in occupations that seem to produce higher numbers of depressed individuals, females may succeed in these occupations more often because they use functional strategies for dealing with emotions more often than males.

We sought to explain a portion of these conflicting findings by focusing on the emotional tones of the interpersonal stressors. Interactions with the public, including all interactions of an employee with various outsiders to the organization (e.g., customers, clients, a government, or the general public), are stressors of neutral tone. Occupational research has identified interactions with the public as emotional stressors for a variety of reasons: first, these interactions are asymmetrical in that the employee’s position is inferior to that of their counterpart; second, these interactions are characterized by short-term personal interactions that are often anonymous and unidirectional; and third, employees are usually required to exhibit positive emotions and hide negative emotions [2], [4]. These factors imply that interactions with the public are stressful because they require emotional work, which may lead to feelings of discomfort. Nonetheless, we suggest that the predominately neutral emotional tone of these interactions allow for individuals to cope with these stressors in functional manners, depending on their resources. We hypothesize that for males, interactions with the public relate positively with rates of depression diagnoses, whereas the relationship may be non-significant or negative for females.

Conversely, conflictual contact is negative in emotional tone and may make functional coping difficult. Therefore, this type of contact should increase the rates of depression in occupations where it occurs frequently. Conflictual contact is characterized by contentious exchanges, hostility, or aggression [28]. Conflictual contact is strongly related to the negative emotions of employees [29]. For some occupations, interactions with outsiders or strangers frequently involve rude or aggressive behaviors [2], [12]. Conflictual contact has been suggested to be particularly harmful to employee wellbeing [4]. Recent research has raised awareness that conflictual contact as a stressor at work impairs functional strategies of emotional coping [30], [31]. Furthermore, beginning with the earliest depression research, it has been suggested that conflict increases internalized negative emotions (e.g., anger) that may lead to depression [32]. Therefore, we see conflictual contact as a strong emotional stressor that increases discomfort for those who work and also restricts the potential functional coping strategies of employees (i.e., our framework is similar to the strong situation hypothesis of [33], [34]). Based on these findings, we further suggest that females’ functional handling of emotional stressors may be advantageous in occupations that involve frequent interactions with the public but not in occupations that involve frequent conflictual contact. We hypothesize that conflictual contact in an occupation relates positively to rates of depression diagnoses.

## Methods

### Data

The analyses used combined data from the German Statutory Pension Insurance (GSPI) agency from 2009 and the Occupational Information Network (O*NET) 17.0 database (status July 2012). The GSPI dataset is available as a scientific use file and can be obtained at http://forschung.deutsche-rentenversicherung.de/ForschPortalWeb/. All information provided by the agency is completely anonymous.

The GSPI is the largest provider of medical rehabilitation benefits in Germany. In 2009, 39.5% of all inpatient medical rehabilitation benefits were financed by the GSPI. This dataset provides a sample of approximately 20% of all statutory pension insurance medical rehabilitation cases in 2009. The sample was randomly drawn by the pension insurance. The data consist of administrative data that were collected for documentation purposes. Among other information, this dataset contains demographic information, medical diagnoses, and most recent occupation. The O*NET database provides detailed descriptions of a total of 900 different occupations within the US workforce, including work descriptions and characteristics. The data were gathered from a variety of sources including job incumbents, supervisors and occupational experts. Such data are currently unavailable in Germany.

In combining these datasets, we adjusted the data as follows: we included only individuals of working age (16–65 years) who were personally insured (rather than being insured through a spouse or child) and had completed one medical rehabilitation measure in 2009 (*n* = 187,936). We then paired every German job title in the GSPI dataset with a counterpart in the O*NET dataset. Two coders used both US job descriptions from the O*NET and job descriptions from the German Federal Employment Agency (inter-rater reliability: Cohen’s *κ* = .81). Occupations that could not be clearly matched with an O*NET occupation were excluded from the pension insurance data (80 occupations, *n* = 22,412). A total of 207 matching occupations were identified. We also dropped cases with missing values for the occupational variable (*n* = 43,064) and cases in which the individual was unemployed (*n* = 24,478). Additionally, we excluded individuals who were not employed full-time (*n* = 19,806) at the time of application for medical rehabilitation based on the argument that the occupational stressors must be sufficiently intense to influence the onset of depression [12]. Additionally, cases without a rehabilitation diagnosis (*n* = 1,551) were excluded from the dataset. Finally, to avoid biases due to small groups, we excluded all occupations for which we found less than 10 cases (12 occupations, *n* = 62). Our final sample contained *n* = 76,563 cases, which were nested into 195 occupations. 63.7% of the individuals were male. The average age was *M* = 48.9 (*SD* = 9.2). In Table 1, we present the means and standard deviations of the study variables for both included and excluded cases.

**Table 1:** Means and standard deviations of study variables.

Variable	M	SD	M	SD	M	SD
	(initial)	(initial)	(dropped)	(dropped)	(final)	(final)
1. Age	49.14	9.41	49.28	9.54	48.94	9.22
2. Gender	.52	.50	.43	.50	.64	.48
3. Trainee	.01	.09	.01	.09	.01	.10
4. Unskilled blue-collar worker	.17	.37	.16	.37	.18	.38
5. Skilled blue-collar worker	.24	.43	.16	.37	.35	.48
6. Foreman	.01	.10	.01	.08	.01	.12
7. White-collar worker/civil servant	.38	.49	.35	.48	.43	.50
8. Self-employed	.03	.16	.04	.19	.01	.09
9. Full-time without shiftwork/piecework/night shift	.43	.49	.20	.40	.74	.44
10. Full-time with shiftwork/piecework	.10	.30	.05	.22	.18	.38
11. Full-time with night shift	.43	.20	.02	.14	.08	.27
12. Depression Diagnosis	.07	.25	.07	.25	.07	.25
13. Proportion of men					.67	.29
14. Conflictual contact					2.43	.49
15. Interactions with the public					2.95	.84

Note. Initial sample *N = *187,936 individuals. Dropped sample *N = *111,373 individuals. Final sample *N = *76,563 individuals in *K = *195 occupations. Age is indicated in years. Variables 2 to 12 are dichotomous, and mean values represent proportions. Gender: 0 = female, 1 = male. Depression diagnosis: yes = 1, no = 0.

### Measures

#### Depression diagnosis

The pension insurance dataset provides rehabilitation diagnoses that were assessed by the attending physician in the rehabilitation hospital according to the criteria of the International Classification of Diseases (ICD), 10^th^ edition. For our analyses, we used the primary diagnosis; i.e., the diagnosis with the greatest importance for the rehabilitation measure that was granted. From this diagnosis, we generated a dichotomous variable that distinguished between ‘depression’ [including the ICD-diagnoses of F32 (depression), F33 (recurrent depressive disorder), and F34 (persistent depressive disorder)] and ‘other diagnoses’. In our sample, 6.8% of the individuals (*n* = 76,563) received a medical rehabilitation measure due to one of these three types of depression. The remaining 93.2% of cases consisted mainly of different somatic diagnoses, such as diseases of the musculoskeletal system or cancer, and a small proportion of other mental diagnoses.

### Individual-Level Covariates

The pension insurance dataset provided *gender* as a covariate. Additionally, a number of control variables could be derived from the dataset including age, work status, and job position. We controlled for *age* as a risk factor because the prevalence of depression increases during working life [35]. We used *work status* to control for different levels of work strain from shift work [36]. This variable distinguishes between “full-time without shiftwork/piecework/night shifts”, “full-time with shiftwork/piecework”, and “full-time with night shifts”. We used *job position* as a proxy for socioeconomic status, which has been related to depression in previous research [37]. The possible job positions were trainee, unskilled blue-collar worker, skilled blue-collar worker, foreman, white-collar worker/civil servant, and self-employed. Both work status and job position were self-reported by the rehabilitation patient and refer to the date of application for rehabilitation, which preceded the initiation of the actual rehabilitation measure (including the diagnosis) by 45 days on average.

### Occupational-Level Covariates

*Interactions with the public* were measured using three O*NET items that assessed the importance of interactions with the public within each occupation [2]. These items were as follows: 1) “deal with external customers – how important is it to work with external customers or the public in this job?”; 2) “communicating with persons outside the organization – communicating with people outside the organization, representing the organization to customers, the public, or the government, and other external sources. This information may be exchanged in person, in writing, by telephone, or by e-mail”; and (3) “performing for or working directly with the public – performing for people or dealing directly with the public. This includes serving customers in restaurants and stores and receiving clients or guests”. The importance levels of these activities within an occupation were rated by job incumbents or occupational experts on a 5-point scale ranging from “Not Important” (1) to “Extremely Important” (5). We combined these three items into an overall score for interactions with the public. The alpha coefficient was *α* = .90.

*Conflictual contact* was measured using three O*NET items that we identified as referring to the amount of conflictual contact entailed by each occupation. These items were as follows: 1) “frequency of situations of conflict – how often does the employee face situations of conflict in this job?”; 2) “dealing with unpleasant or angry people – how frequently is the worker required to deal with unpleasant, angry, or discourteous individuals as a part of their job?”; and (3) “deal with physically aggressive people – how frequently does this job require the worker to deal with the physical aggression of violent individuals?” The frequencies of these situations were rated by job incumbents or occupational experts for each occupation on a 5-point scale, which ranged from “Rarely - includes once a year or less” (1) to “Frequently - includes daily, several times a day, hourly or more” (5). These three items were combined into an overall conflictual contact score. The alpha coefficient for this scale was *α* = .85.

As a control variable at the occupational level, we used the *proportion of men* in each occupation, which was provided by the 2009 employment statistics of the German Federal Employment Agency, to indicate whether each occupation was predominantly entered by males or females across the general population. We included this variable to protect against ecological fallacies in the cross-level interaction effects [38].

### Statistical Analyses

It is our goal for this paper to investigate contextual effects of the occupational environment on individuals who work in these occupations [39]. Multilevel regression modeling is a methodology for analyzing data with a focus on the decomposition of variance in nested data, for example for data concerning employees in occupations [40], [41]. We use logistic regression due to the dichotomous nature of our outcome (depression diagnosis yes/no). By considering the nested structure of the data, multilevel logistic regression modeling allows us to separately investigate relationships between individuals and of occupations, as well as interactions between both levels on individuals’ depression diagnoses. Analyses were done using STATA (version 12). Variables were included in the final model based on theoretic considerations.

## Results

The correlations between the study variables and the descriptive statistics are illustrated in Table 2.

**Table 2:** Pearson Correlations between Variables at the Individual (below the diagonal) and Occupational Levels (above the diagonal).

Variable	1	2	3	4	5	6	7	8	9	10	11	12	13	14	15
1. Age															
2. Gender	.04***														
3. Trainee	−.24***	−.02***													
4. Unskilled blue-collar worker	.04***	−.01**	−.05***												
5. Skilled blue-collar worker	.01**	.29***	−.07***	−.35***											
6. Foreman	.02***	.07***	−.01**	−.06***	−.09***										
7. White-collar worker/civil servant	.01	−.31***	−.09***	−.41***	−.65***	−.10***									
8. Self-employed	.00	.02***	−.01**	−.05***	−.07***	−.01**	−.08***								
9. Full-time without shiftwork/piecework/night shift	.04***	.01***	.03***	−.16***	.00	.01***	.11***	.03***							
10. Full-time with shiftwork/piecework	−.03***	.01***	−.02***	.14***	.00	−.01***	−.10***	−.03***	−.80***						
11. Full-time with night shift	−.02***	.04***	−.02***	.06***	.00	.00	−.04***	−.01***	−.49***	−.13***					
12. Depression Diagnosis	−.03***	−.13***	−.00	−.01	−.07***	−.01	.08***	−.01***	−.01***	.01***	−.00		−.66***	.20**	.33***
13. Proportion of men														−.24***	−.46***
14. Conflictual contact														.*85*	.47***
15. Interactions with the public															.*90*

*Note. N = *76,563 individuals in *K = *195 occupations.

Age is indicated in years. Gender: 0 = female, 1 = male. Depression diagnosis: yes = 1; no = 0. The reliabilities (i.e., the α coefficients) of the scales are italicized in the diagonal where applicable. *p<.05. **p<.01. ***p<.001.

Regarding individuals’ risks of depression diagnoses, 8.4% of the variation in depression diagnoses could be attributed to occupational level (Null Model, Table 3).

**Table 3:** Results of Cross-Level Analyses to Predict Depression Diagnoses in Full-Time Employees.

Variable	Null model	Model 1	Model 2	Model 3
	*OR* [95% CI]	*OR* [95% CI]	*OR* [95% CI]	.*OR* [95% CI]
Individual				
Intercept/constant	.06 [.05,.07]	.08 [.06,.12]	.08 [.06,.11]	.11 [.08,.16]
Gender		.55 [.51,.59]	.47 [.41,.53]	.21 [.13,.33]
Age (centered)		.99 [.98,.99]	.99 [.98,.99]	.99 [.98,.99]
Occupational status				
Skilled blue-collar worker				
Trainee		.61 [.44,.85]	.61 [.43,.85]	.61 [.43,.85]
Unskilled blue-collar worker		1.26 [1.14, 1.38]	1.23 [1.12, 1.35]	1.22 [1.11, 1.34]
Foreman		1.48 [1.13, 1.94]	1.50 [1.14, 1.97]	1.50 [1.14, 1.97]
White-collar worker/civil servant		1.20 [1.10, 1.31]	1.19 [1.09, 1.30]	1.19 [1.09, 1.30]
Self-employed		.62 [.41,.95]	.62 [.41,.95]	.62 [.40,.94]
Scope of work				
Full-time without shiftwork/piecework/night shift				
Full-time with shiftwork/piecework		1.14 [1.05, 1.23]	1.13 [1.04, 1.22]	1.14 [1.05, 1.23]
Full-time with night shift		1.17 [1.04, 1.31]	1.16 [1.04, 1.30]	1.17 [1.04, 1.31]
Occupational				
Conflictual contact		1.14 [1.00, 1.30]	1.15 [1.02, 1.29]	1.12 [.97, 1.28]
Interactions with the public		1.02 [.93, 1.11]	1.02 [.93, 1.11]	.94 [.85, 1.04]
Proportion of men		.49 [.39,.61]	.62 [.49,.78]	.63 [.50,.79]
Interaction Terms				
Gender*Conflictual contact				1,09 [.88, 1.35]
Gender*Interactions with the public				1.21 [1.05, 1.39]
Random effects	*Est.* [95% CI]	*Est.* [95% CI]	*Est.* [95% CI]	*Est.* [95% CI]
Intercept SD	.55 [.06,.07]	.22 [.16,.29]	.18 [.11,.29]	.16 [.10,.27]
Slope (Gender) SD			.35 [.24,.49]	.28 [.17,.44]
Intercept-Slope Correlation			−.38 [−.77,.19]	−.12 [−.69,.54]
ICC	.08			
Log Likelihood	−18443.58	−18109.09	−18096.70	−18089.47
LR-Test				

*Note.* Occupations were included if *n*>10. The largest group included *n* = 8,740 individuals. *OR* = odds ratio; values below 1 indicate reduced, and values above 1 indicate increased depression diagnoses. 95% Confidence Intervals (CI) indicate the significance of these analyses if 1 is excluded (OR) or if 0 is excluded (Estimates in the random effects part of the table).

The results from a random intercept model (Model 1, Table 3) that included all covariates and control variables in which the intercept was allowed to vary randomly across occupations revealed an intercept of *OR* = .08 and a standard deviation of the associated random effect of *SD* = .22 (Model 1, Table 3). This result indicates that a proportion of 7% of all diagnoses were depression diagnoses (Proportions are calculated *prop* = *OR/1+OR* [38]), and the average risk for a diagnosis of depression compared to other diagnoses varies across occupations. Moreover, these results indicate that the males had a reduced risk of depression diagnosis compared to the females (1 = male, 0 = female; *OR* = .55, *p*<.001) – men were diagnosed with a proportion of 36% of all depression diagnoses. In occupations with high conflictual contact there was a slightly elevated risk of depression diagnoses (*OR* = 1.14, *p*<.05). For individual level control variables, odds ratios below one for the occupational groups “trainee” and “self-employed” indicate reduced depression rates in these occupational groups compared with skilled blue collar workers, whereas all other occupational status groups show increased depression rates. Considering scope of work, both shiftwork with and without night shift increase depression rates compared with work without shift work. For the proportion of males in an occupation as an occupational level control variable, results indicate that rates of depression diagnoses are reduced in occupations with high male labor force.

Using a random slope model (Model 2, Table 3), we investigated whether the gender effects on the relative risk for a depression diagnosis varied randomly between occupational groups compared to other diagnoses. The results revealed considerable slope differences between occupations with a standard deviation of the slopes of gender between occupations of *SD = *.35 (*p*<.05). Ninety-five percent of the gender slopes ranged between *OR* = .23 (indicating strong differences between male and female slopes) and *OR* = .93 (indicating little differences, calculated and exponentiated from logit values and standard deviations). These results indicate that – if at all – only few occupations exist in which males have higher proportion of depression diagnoses than females, but nonetheless, there is large variation. The negative correlation between slope and intercept in the random effects model indicates that occupations with higher rates of depression diagnoses (irrespective of gender) also tend to have more similar slopes for individuals of both genders. The random slope model produced an improved model fit (Likelihood ratio test: *Chi^2^* = 24.79, DF = 1; *p*<.001) compared to that of the random intercept model.

**Figure 1:**
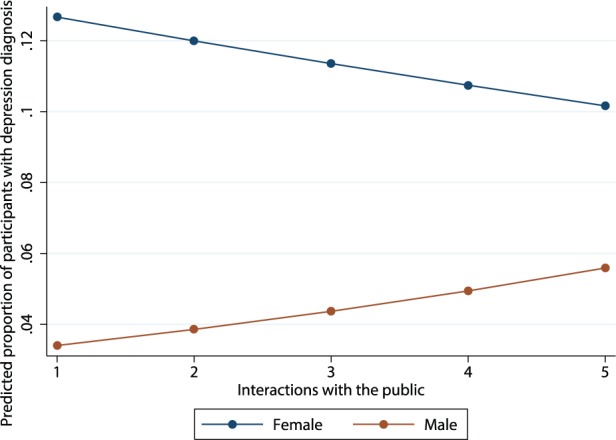
Cross-Level Interaction Plot of Gender and the Occupations’ Interactions with the Public.

Finally, we specified cross-level interaction effects between gender and the occupational-level variables of conflictual contact and interactions with the public to test the extent to which the different effects of gender across occupations depended on these occupational attributes. The results (Model 3, Table 3) revealed a positive influence of the interaction between gender and interactions with the public on depression diagnoses (*OR* = 1.21, *p*<.01). We plotted this effect in Figure 1. The plot shows that the relationship between interactions with the public and depression diagnoses is negative among women but positive among men. These results extend our interpretation from model 2. To inspect the nature of the interaction effect we calculated odds for men compared with women to be diagnosed with depression at various degrees of interactions with the public. As expected, odds for men compared with women were significantly lower in occupations characterized by low and medium interactions with the public (low: *OR* = .25, *p*<.01, medium: *OR* = .37, *p*<.01), but not in occupations characterized by high interactions with the public (*OR* = .55, *p* = .11). This indicates that slopes of both genders are rather similar in occupations characterized by high interactions with the public but dissimilar in occupations characterized by low interactions with the public. The interaction effect of gender and conflictual contact was non-significant. The random slope model that includes the cross-level interaction terms produced an improved model fit (Likelihood ratio test: *Chi^2^* = 14.46, DF = 2; *p*<.001) compared with the random slope model.

## Discussion

Together, our results support suppositions concerning the ‘hell is other people’ [1] effect in occupational environments. We found that (1) a considerable portion of the variance in individual depression diagnoses resides between occupations, which indicates that occupations are relevant for individuals’ likelihoods of becoming depressed; 2) an increased frequency of depression diagnoses among females compared to males across occupations [10]; 3) individual level variations in the relationship of gender and depression between occupations, which indicates that the likelihoods with which males and females develop depression rather than other disorders vary across occupations. We investigated the reasons for this result and found (4) a significant moderating effect of interactions with the public on the gender–depression relationship (Figure 1). Our results further indicate that the amount of conflictual contact in occupations was related to increased occurrences of depression diagnoses in our rehabilitation dataset. The increased odds for clinically diagnosed depression related to conflictual contact were significant, but small for this dataset. However, patients need to pass a high threshold to be treated with medical rehabilitation due to depression. Larger effects may be found for dependent variables with a lower threshold, for example for symptoms of depression instead of full-blown depression. In sum, we have provided a differentiated picture of the ‘hell is other people’ effect.

We interpret our findings as follows. First, our results support the notion that both the tone of interactions with strangers and the gender-specific handling of such interactions may play roles in determining whether strangers constitute a risk factor for the development of depression. Second, in accordance with the job demand-resources model [42], we argue that certain resources are needed to fulfill the demands placed on an individual by interactions with the public. Here, we draw from previous research, which argues that occupations characterized by low interaction with the public require instrumental resources such as advice, contracts, or challenging and visible assignments to provide career direction and promotions [43], whereas occupations characterized by frequent interaction with the public require socio-emotional resources, such as emotional intelligence, emotion regulation ability and emotional expressiveness [44]. Other researchers have detected gender differences with respect to these resources (see Table 4). These arguments may, at least partially, explain our finding of interaction effects. However, we note that the effects are small, perhaps because gender differences are small [43]. Third, we suggest that the results concerning conflictual contact identify conflictual contact as a strong situational stressor. Here, differences between genders (for example, in their resources for handling emotions) do not play a role. We interpret this finding in line with the strong situation hypothesis [34] in suggesting that the conflictual situation does not leave room for gender-specific strategies. Fourth, we suggest that additional moderators (for example, economic indicators of resource inequality) may influence the relationship between gender and depression diagnoses. Such analyses are beyond the scope of this work. However, the results show that relationships between occupational status and depression diagnoses are higher among regular employees than among, for example, the self-employed.

**Table 4:** Interpretation of the interaction finding in a demand-resource-congruence framework.

	Interactions with the public	
	*Low*	*high*
Exemplaryoccupations^a^	All sorts of manufacturing suchas tire builders (197)or rock splitters^+^ (180), furnace,kiln, oven, drier, and kettleoperators and tenders* (195),locomotive engineers (155), cooksin a restaurant (149), mathematicians^+^(140), or chemists (138)	Police patrol officers* (1), sales agents (travel, real estate, insurance), healthcare social workers* (7), reporters (11), hairdressers (13), registered nurses* (27), actors^+^ (38), or chief executives* (45)
Occupation-specificstressors	Stressors from workorganization, repetition,decision latitude, etc.	Emotional stressors
Necessary resources forhandling thesestressors	Individual behavioral strategiessuch as individualagency, direct action andassertiveness [19].	Interpersonal emotion focused behavioral strategies such as withdrawal, cautious or indirect action, or compromise [19].
	Instrumental resources such asadvice networks, contracts, orchallenging and visible assignments(for career direction andpromotions) [43].	Socio-emotional resources such as emotional intelligence, emotion regulation ability and emotional expressiveness (for reflection, assistance and guidance) [44].
Gender-specificresources	Studies indicate higher individualbehavioral strategies[19] and individual instrumentalresources, for examplerelationships with higher statusindividuals, within mencompared with women [43], [51].	Studies indicate higher levels of interpersonal behavioral strategies and socio-emotional resources within women compared with men [17], [20], [27], [52], but also repressed emotionality as a negative resource within men compared with women [18], [19].
Congruence ofOccupationspecific stressorsand genderspecific resources[44], [53]–[55]	*High congruence within* *males, low congruence* *within females*	*Low congruence within males, high congruence within females*

*Note.* ^a^ *K = *197 occupations in the dataset. These were ranked according to amount of interaction with the public; low/high numbers indicate low/high interaction with the public. As an illustration, we additionally indicate the amount of conflictual contact in an occupation if it is especially high (*) or low (^+^).

In the present study, we were able to overcome a number of limitations that we have observed in current studies in the field. First, occupational depression research is often limited to service occupations, for example, waiters, bus drivers, doctors, etc. (e.g., [5], [15], [25], [45], [46]), and neglects problems in other occupations [3]. In contrast, broader, population-wide studies of the prevalence of depression often neglect the influences of occupation on the onset of depression (e.g., [47]). Second, the value of many studies of the effects of the workplace on the onset of depression is limited because these studies often refer to depressive symptoms, sub-syndromal depression, or reduced well-being, and not clinical depression per se, as an outcome because those with major depression are frequently not working. Furthermore, the vast majority of studies have exclusively used self-report data, which may lead to (selective) underreporting of depressive episodes and other problems (see [48] for the argument). Finally, regarding gender differences in the antecedents of clinical depression, our study showed that the differential/unequal influences of the work environment on both genders are understudied [10].

Our dataset and analyses provide this study with a few specific strengths. First, our dataset covered a wide range of occupations and was less selective than the datasets produced by most data collection efforts of individual scholars. Second, in contrast to other studies (e.g., [48], this study did not rely on questionnaire–based retrospective answers provided by respondents; rather, at the individual level, our dataset solely drew from routine data that was not collected specifically for analyses. Third, with a sample size of over 70,000 individuals from 196 occupations, the sample size of this study was larger than those of most other studies in the field. Recently, Muchinsky and Raines [49] offered the criticism that, of the large number of occupations that exist in economic systems, only a few are regularly studied by researchers.

This study has a number of limitations. First and most importantly, the dataset was comprised solely of individuals who underwent rehabilitation in 2009; thus, this dataset excluded not only healthy individuals but also those who suffered from depression without entering rehabilitation. Given this limitation, we cannot conclude that the elevated rates of rehabilitation due to depression within specific occupations are related to the rates of becoming sick within those occupations. Second, the data analyzed here stem from multiple sources, which reduces common method and source biases but is also accompanied by the drawbacks that there may have been differences in the timing of data collection and occupational characteristics between datasets. Third, we used routine data which frees the data from biases due to the researchers; however, some potentially interesting information was not collected. For example, we do not know the durations with which the subjects had worked in their occupations prior to rehabilitation, and we cannot control for the conscious or unconscious self-selection of individuals into certain occupations with higher or lower risks of depression onset due to the individuals’ vulnerability to depression. Fourth, we were unable to investigate whether self-selection of vulnerable individuals into certain occupations influenced the results. Fifth, individual occupations may differ from the occupational characteristics described in the O*Net dataset due to multiple roles that employees may have in an organization.

This study has practical implications because full remission after depression is difficult to achieve, and residual sub-threshold symptoms increase the likelihood of chronic depression [50]. Thorough knowledge of the factors that increase the risk of depression may therefore be important for both employers and employees. We suggest that individuals may focus on their resources and compare these to typical occupational stressors. For example, this knowledge could be beneficial to many western societies that are dealing with demographic changes and shortages of skilled professionals. In Germany, shortages of specialized laborers in a number of occupations are expected, and these occupations include those that were found to be related to greater risks of depression (e.g. registered nurses). For employers of employees in occupations with high risks of depression diagnoses, these results may provide an impetus to build the emotional resources that help employees cope with these stressors [19].
